# Pharmacogenomic and epigenomic approaches to untangle the enigma of IL-10 blockade in oncology

**DOI:** 10.1017/erm.2023.26

**Published:** 2024-01-08

**Authors:** Noha M. Elemam, Radwa Y. Mekky, Gowhar Rashid, Maria Braoudaki, Rana A. Youness

**Affiliations:** 1 Research Instiute for Medical and Health Sciences, University of Sharjah, Sharjah, United Arab Emirates; 2 Clinical Sciences Department, College of Medicine, University of Sharjah, Sharjah, United Arab Emirates; 3 Department of Pharmacology and Toxicology, Faculty of Pharmacy, October University for Modern Sciences and Arts (MSA University), Cairo 12622, Egypt; 4 Amity Medical School, Amity University, Gurugram (Manesar) 122413, Haryana, India; 5 Department of Clinical, Pharmaceutical and Biological Sciences, School of Life and Medical Sciences, University of Hertfordshire, Hatfield AL10 9AB, UK; 6 Biology and Biochemistry Department, Faculty of Biotechnology, German International University, Cairo 11835, Egypt

**Keywords:** Cancer, Cytokines, IL-10, Interleukins, Non-coding RNAs, Pharmacogenomics, Tumour-microenvironment

## Abstract

The host immune system status remains an unresolved mystery among several malignancies. An immune-compromised state or smart immune-surveillance tactics orchestrated by cancer cells are the primary cause of cancer invasion and metastasis. Taking a closer look at the tumour-immune microenvironment, a complex network and crosstalk between infiltrating immune cells and cancer cells mediated by cytokines, chemokines, exosomal mediators and shed ligands are present. Cytokines such as interleukins can influence all components of the tumour microenvironment (TME), consequently promoting or suppressing tumour invasion based on their secreting source. Interleukin-10 (IL-10) is an interlocked cytokine that has been associated with several types of malignancies and proved to have paradoxical effects. IL-10 has multiple functions on cellular and non-cellular components within the TME. In this review, the authors shed the light on the regulatory role of IL-10 in the TME of several malignant contexts. Moreover, detailed epigenomic and pharmacogenomic approaches for the regulation of IL-10 were presented and discussed.

## Introduction

The use of immunotherapy as a novel therapeutic approach in preventing cancer has become widespread (Ref. 1). Immune checkpoint blockade modalities targeting PD-1 and CTLA-4 provide long-lasting immune responses with established therapeutic benefits for some cancer patients (Refs 2–6). Although, targeting cytokines is considered a crucial approach in immunotherapy as evidenced in the treatment of solid tumours, such as renal cell carcinoma (RCC) and melanoma, only interferons (IFNs) and IL-2 have been approved by Food and Drug Administration (FDA) for use as cancer therapies (Ref. 7).

IL-10 is considered one of the very promising targets for immunotherapy; however, its controversial role in carcinogenesis hinders the applicability of benefiting from its blockade in cancer treatment (Ref. 8). IL-10 has been shown to possess both anti- and pro-inflammatory roles in cancer (Ref. 9). The intensity of the immunological response to both self and foreign antigens is reduced by IL-10. In light of this, IL-10 signalling blockage improves vaccine-induced T-cell responses and tumour growth inhibition (Ref. 10). On the other hand, tumour regression is also induced by exogenous IL-10, particularly PEGylated (PEG)-IL-10 (Ref. 11). This paradoxical data urges the need to investigate the role of pharmacogenomics, epigenetics and genetic variants in IL-10 and its receptor to identify those patients that might benefit from IL-10 targeted therapies. In this review, the authors will address the role of IL-10 in cancer, the currently available IL-10-based immunotherapy, the epigenetic regulation of IL-10 and the single nucleotide polymorphisms (SNPs) present in IL-10 that might influence patient responses to therapy.

## The tumour microenvironment

**Figure 1.**
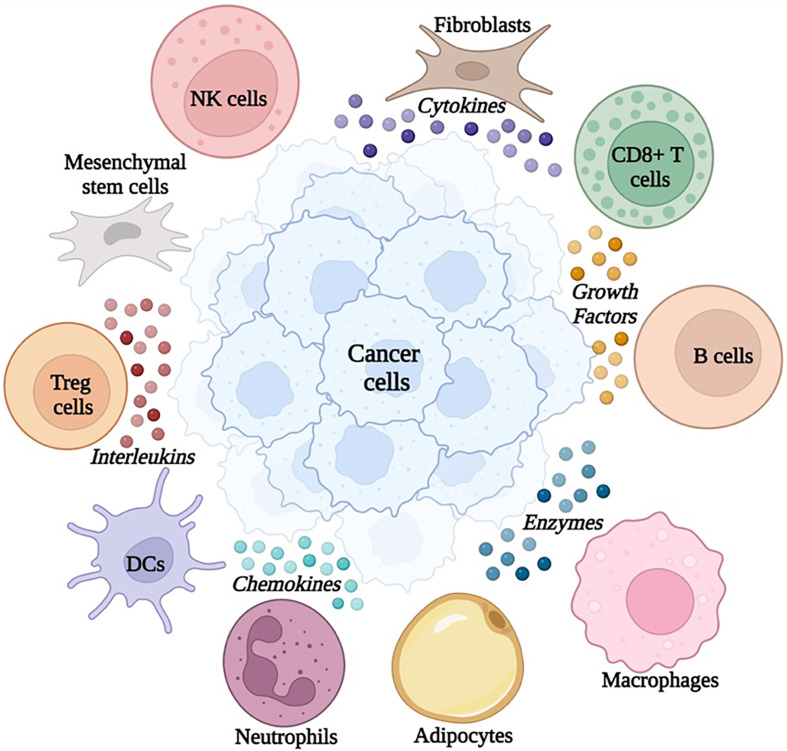
Snapshot of cellular and non-cellular components of the tumour microenvironment

Cancer definition has been revolutionized over the past few decades from the concept of being abnormal cells to a plethora of complex network that is made up of both neoplastic cells with their surrounding stroma (Refs 1, 4, 6, 12). The multifaceted dynamic milieu of cellular components along with non-cellular compartments portrays what is now known as the tumour microenvironment (TME) (Refs 6, 13, 14). Such a microenvironment could control the aggressiveness, rate of growth and metastatic potential of the tumour (Refs 15–18). These cellular components include immune cells such as T lymphocytes (Refs 19–24), regulatory T cells (Tregs) (Ref. 25), B lymphocytes, natural killer (NK) cells (Refs 16, 26–29), mesenchymal stem cells (Refs 30, 31), tumour-associated-macrophages (Refs 32, 33), tumour-associated neutrophils (Refs 34, 35), dendritic cells (DCs) (Ref. 36) and non-immune cells such as pericytes (Ref. 37), adipocytes (Refs 38, 39), myeloid-derived suppressor cells (MDSCs) (Refs 40–42) and cancer-associated fibroblastic cells (Refs 43, 44). Interestingly, these immune cells drive the production of soluble components that include cytokines, chemokines, growth factors and extra-cellular remodelling enzymes (Refs 27, 28). Such mediators, particularly cytokines, assist in the communication between the cellular TME components and cancer cells as shown in Figure 1 (Refs 45, 46).

### Interleukin-10 (IL-10)

One of these cytokines is the paradoxical interleukin ‘IL-10’, which remains an integral part of several malignancies, and regulates the secretion of other cytokines. This pleiotropic cytokine was characterized early in the late 1980s and was named cytokine synthesis inhibitory factor (Refs 47, 48). Later on, six immune mediators (IL-10, IL-19, IL-20, IL-22, IL-24 and IL-26) were grouped into the IL-10 family of cytokines based on their similarities with respect to the structure and location of their encoding genes, their primary and secondary protein structures and the receptor complexes (Refs 49–51). Out of these six members, IL-10 has been recognized as a major member mediating different functions within the immune system and cancer cells (Ref. 52).

### Paradoxical role of IL-10 in oncology

#### IL-10 produced by immune cells

IL-10 has also been causally linked to immunity in both the innate and adaptive immune arms. Different triggers have been shown to induce IL-10 production in various immune cells (Ref. 53). The main source of IL-10 appears to be monocytes, and different T-cell subsets (Ref. 54). Moreover, DCs, B cells, NK cells, mast cells, as well as neutrophils, and eosinophils can also synthesize IL-10 (Ref. 54). During infection, macrophages are considered a major source of IL-10. Several toll-like receptors (TLRs), including TLR2, TLR4, TLR5, TLR7 and TLR9 have been shown to induce IL-10 production in macrophages and DCs (Refs 55–63). Also, IL-10 production in DCs is enhanced by the co-activation of TLR2 and Dectin-1 (Ref. 64). Following exposure to IL-10, DCs can initiate the development of regulatory T cells (Tregs) that limit these effector responses (Refs 65, 66). B cells also express several TLRs which have been shown to promote IL-10 production including TLR2, TLR4 or TLR9 (Refs 67–69). Nonetheless, it is also worth mentioning that IFN-*α* augments IL-10 production if combined with TLR agonists from B cells (Refs 70, 71). Additionally, neutrophils produce IL-10 in response to TLR and C-type lectin co-activation through myeloid differentiation primary response 88 (MyD88) and spleen tyrosine kinase (SYK), respectively (Ref. 72).

The key producer of IL-10 is Treg cells that produce other immunoregulatory cytokines, such as TGF-*β* (Ref. 73). The production and action of both cytokines IL-10 and TGF-*β* are involved in a positive feedback loop (Ref. 74). Concerning the mechanism of IL-10 production from Tregs, it has been shown that IL-2 and IL-4 induce IL-10 production from Tregs (Refs 75–77). Additionally, a study concluded that TGF-*β* is required for the differentiation and production of IL-10 from Tregs (Ref. 78). IL-2 and IL-27 are responsible for inducing IL-10 expression in cytotoxic CD8^+^ T cells (Ref. 79). However, IL-12 and IL-23 prime CD8^+^ and CD4^+^ T cells for IL-10 production (Refs 80–82).

Some studies reported IL-10 immunosuppressive effects such as inhibiting IFN-*γ* and TNF-*α* production by NK cells *in-vitro* (Ref. 83). However, other studies reported IL-10 immunostimulatory effects via the promotion of NK cell cytotoxicity in preclinical models (Refs 9, 84). Adding to the complexity of this master cytokine, one of the studies has shown that the exposure of malignant cells to IL-10 resulted in a reduction in their sensitivity to cytotoxic T cells but an increase in NK cell cytotoxicity (Ref. 85). This might suggest that IL-10 contributes to fighting malignant cells by stimulating the immune innate arm (Ref. 86).

As mentioned earlier, one of the main drivers of IL-10 expression in many immune cells is TLR signalling (Ref. 56). TLR ligation leads to the activation of several downstream pathways, including the mitogen-activated protein kinase (MAPK) pathway and the phosphoinositide 3-kinases (PI3K)/AKT pathways (Ref. 87). Activation of the MAPK and downstream extracellular-signal-regulated kinase (ERK1 and ERK2) are critical for IL-10 production in macrophages and DCs in response to several TLR activators (Refs 58, 62, 88, 89). The MAPK pathway eventually results in the activation of several transcription family members such as the activator protein-1 (AP-1) which activates IL-10 transcription (Refs 55, 58, 62, 90). Moreover, ERK and p38 also contribute to IL-10 production in TLR-stimulated macrophages, monocytes, and DCs (Refs 57, 89–92). Both ERK and p38 may function cooperatively in their regulation of IL-10 production, through their joined activation of mitogen and stress-activated protein kinases (MSK1 and MSK2) which promote IL-10 production in TLR-stimulated macrophages. Downstream of MSK1 and MSK2 are the transcription factors, cAMP-response element binding protein (CREB), and AP-1, which also bind and transactivate the IL-10 promoter (Refs 93–95). Moreover, it is worth mentioning that both ERK and p38 were shown also to directly phosphorylate Sp1, one of the IL-10 transcription factors (Refs 96, 97).

The phosphatidylinositol-3-kinase (PI3K/AKT) pathway also contributes to IL-10 expression in myeloid cells either by antagonizing glycogen synthase kinase 3 beta (GSK3-*β*), a constitutively active kinase that inhibits the production of IL-10 or through ERK and mammalian target of rapamycin (mTOR) and STAT-3 activation (Refs 98–100).

#### IL-10 produced by cancer cells

**Figure 2.**
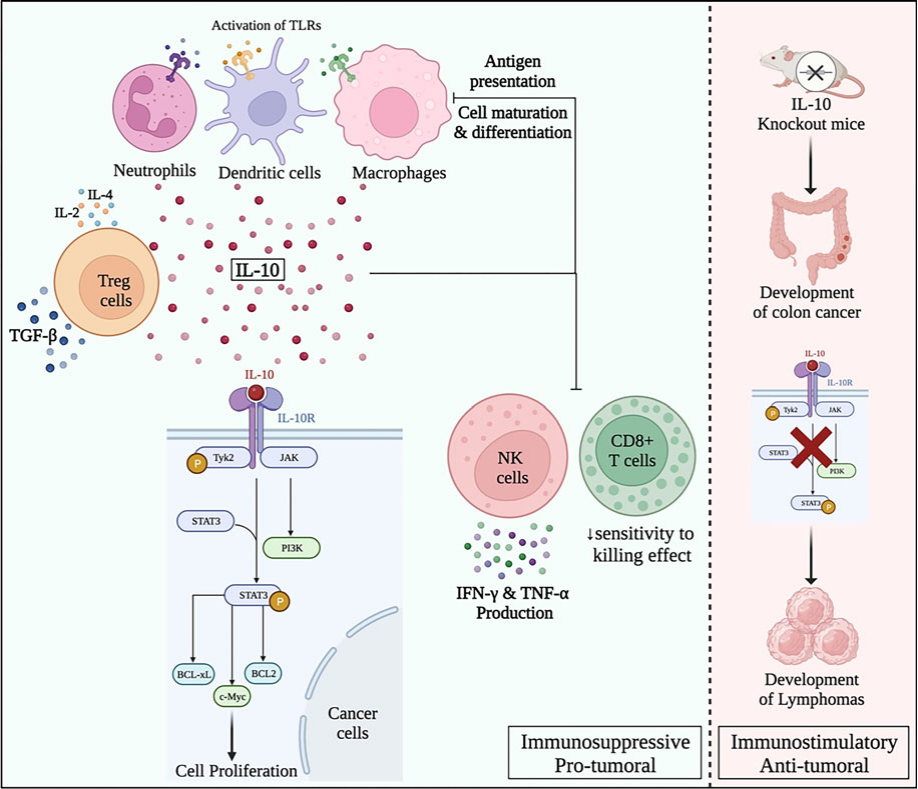
Paradoxical pro- and anti-tumour roles of IL-10 in oncology

IL-10 has been linked to many types of cancers such as gastric cancer (Ref. 101), cervical cancer (Ref. 102), lung cancer (Ref. 103), breast cancer (Ref. 104), colon adenocarcinoma (Ref. 105), head and neck cancer (Ref. 106), oesophageal cancer, nasopharyngeal cancer, oral cancer (Ref. 107) and colorectal cancer (Ref. 108). Its role in tumourigenesis is reported to be controversial where it could be a tumour suppressor or promoter. However, due to the complex nature of IL-10, its role in shaping the TME remains a gap that needs further research. Most of the literature is directed towards presenting the pro-tumoural activity of IL-10 in different oncological settings. This could be through the positive feedback loop with STAT-3, as IL-10 has been shown to activate STAT-3 resulting in the upregulation of B-cell lymphoma 2 (BCL-2) or B-cell lymphoma-extra-large (BCL-xL), and stimulation of cell proliferation by cyclins D1, D2, B, and proto-oncogene *c-Myc*, thus contributing to cancer progression (Ref. 93). On the other hand, IL-10 immunosuppressive activity has been reported on macrophages and DCs, where it was found to dampen antigen presentation, cell maturation, and differentiation resulting in tumour immune evasion as shown in Figure 2 (Ref. 109). Several studies have examined the role of IL-10 in different types of malignancies as listed in Table 1 below.

**Table 1.** Role of IL-10 in different solid malignancies

Cancer type	Effect of IL-10	Reference
Colon cancer	Serum level of IL-10 was correlated with reduced cytotoxic activity of CD8 + T cells in MC38- mouse colon cancer model	(Ref. 110)
Gastric cancer	Induction of the autocrine secretion of IL-10 from DCs resulted in the maturation of DCs yet the antigen delivery activity was inhibited, leading to evasion of the host immune surveillance and the development of gastric cancer.	(Ref. 111)
Breast cancer	Neutralizing IL-10 using anti-IL-10 antibody resulted in the attenuation of STAT3 activation and decreased Bcl-2 mRNA expression and reduced BC cells chemoresistance	(Refs 112, 113)
Ovarian cancer	Recombinant IL-10 enhanced cellular migration verifying the pro-tumoural activities of IL-10 in ovarian cancer.	(Ref. 114)
Bladder cancer	Paracrine secretion of IL-10 by bladder cancer cells was found to paralyse most of the host immune-regulatory actions to concur the tumour.	(Ref. 115)
Cervical cancer	Evaluation of IL-10 expression in cervical lesions, IL-10 mRNA was detected positive only in precancerous and invasive cervical cancers. None of the patients with normal cervical cytology expressed the IL-10 mRNA. IL-10 was also shown to contribute to human Papillomavirus persistency to establish a low-grade squamous intraepithelial neoplasia (LGSIL), then high-grade squamous intraepithelial neoplasia (HGSIL) and finally, progression to cervical cancer.	(Ref. 116)

Previous studies highlighted a significant correlation between IL-10 and the percentage of plasma cells in multiple myeloma patients as it induces the proliferation of plasma cells (Refs 117–119). Other studies indicated an elevation of IL-10 in different haematological malignancies such as Hodgkin lymphoma and non-Hodgkin lymphoma (Refs 120, 121). High IL-10 levels were reported to be associated with a shorter survival rate among patients with diffuse large-cell lymphoma (Ref. 120). Similarly, high IL-10 levels was found to be a prognostic factor in peripheral T cell lymphoma, which can lead to worsening of overall survival, low complete response rate, and higher early relapse rate (Ref. 122). Moreover, elevated IL-10 at diagnosis was found to be an independent prognostic marker in adult hemophagocytic lymphohistiocytosis patients in order to find the right treatment strategy (Ref. 123).

#### The riddle of IL-10 at the tumour-immune cell synapse

The balance between pro-inflammatory and anti-inflammatory signals is generally crucial for the maintenance of normal physiology and the prevention of cancer and a wide variety of diseases (Refs 14, 124–126). In the context of IL-10, it plays a dual function acting either as a pro-inflammatory or an anti-inflammatory mediator (Ref. 127). Regarding its role in cancer, studies have reported that IL-10, secreted by tumours or tumour-infiltrating immune cells, has allowed malignant cells to escape from the immune surveillance (Refs 128–130). In a study by Neven *et al*., IL-10 knockout in mice promoted the development of colon cancer. Moreover, the same study showed that humans deficient in IL-10 signalling molecules were more prone to develop lymphomas at a younger age (Ref. 131). As an anti-inflammatory cytokine, IL-10 is considered crucial for the homoeostasis of the anti-inflammatory Tregs and the suppression of proinflammatory IL-17-expressing T cells. However, IL-10 action depends on multiple factors such as targeted cells, other stimuli, and the time and duration of its effect (Ref. 132). Though, with many rationales presented, a question mark continues to rise to explore the nature of this complex cytokine.

### Is IL-10 blockade a possible option as a novel immunotherapeutic approach for cancer patients?

Controversial data exists regarding the effectiveness of IL-10 immunotherapy in cancer (Ref. 133). Cancer vaccines that utilized monoclonal antibody (mAb) against IL-10 receptors succeeded to increase CD8^+^ T cell responses and to inhibit tumour growth whether injected intraperitoneally or subcutaneously (Refs 134, 135). The beneficial effect of IL-10 blockade is best explained through the inhibition of IL-10-induced suppression of DCs and prevention of their antigen presentation capacity by decreasing the expression of MHC class II and co-stimulator molecules (Ref. 136). Thus, DC-based vaccinations that disrupt IL-10 signalling provide more potent anti-tumour responses (Ref. 136). On the contrary, others claimed that antibodies targeting IL-10R had no protective effect against tumour growth when used with vaccines containing adjuvants that do not induce IL-10, such as the TLR3 ligand poly (I: C) or anti-CD40 agonistic antibodies (Ref. 137). Such a controversy regarding the effectiveness of therapeutic immunization could be explained and summarized by vaccine-induced IL-10 rather than IL-10 produced by tumours (Ref. 137).

It was previously reported that the prognosis of cancer patients is inversely correlated with elevated serum and tumour IL-10 levels (Ref. 138). Despite that, exogenous administration of IL-10 was tested in clinical studies, and resulted in immunological activation, as evidenced by higher granzymes and IFN in the serum of those patients receiving treatment. Pegylated recombinant (PEG) murine IL-10 promoted rejection of tumours and metastases by enhancing CD8 + T cell-mediated immune responses (Ref. 139). In addition, PEG-IL-10 exhibited immunologic and clinical advantages in solid tumours in clinical trials, particularly in RCC and uveal melanoma (Ref. 140). CD8 + tumour-infiltrating lymphocytes (TILs) in metastatic melanoma co-upregulate IL-10R and PD-1. While PD-1 blockade or IL-10 neutralization as monotherapies were insufficient to produce anti-tumour activity, combination therapies of PD-L1 blockers with IL-10R blockers were shown to exert anti-tumour effects by enhancing T cell responses, thereby suppressing the tumour growth (Ref. 141). Similarly, mice with ovarian tumours treated with PD-1 blocking antibodies have higher levels of IL-10 in their serum and ascites. Moreover, infiltration of immunosuppressive MDSCs was reduced, and the immunological activity was increased when IL-10 and PD-1 blockers were used together (Ref. 142). On the other hand, a multi-centred trial involving 111 patients with advanced malignant solid tumours unresponsive to previous therapies revealed that anti-PD-1 treatment (pembrolizumab or nivolumab) in combination with PEG-IL-10 offered a new therapeutic option (Ref. 143).

Most of the immune cells express IL-10 receptors and can activate subsequent downstream signalling pathways. Therefore, the paradox underlying the IL-10 blockade and whether it carries a beneficial or detrimental role in cancer treatment might be deciphered if we understood how exactly these cells react to IL-10 signalling through comprehensive genomic, epigenomic, and proteomic analysis.

### Epigenomic approach

Epigenetic regulations include DNA methylation, histone modifications, histone acetylation, and the action of non-coding RNAs (ncRNAs) (Refs 144–146). Epigenetics arising from an alteration in the chromatin usually leads to alterations in gene expression. Moreover, epigenetic changes could either activate or suppress an oncogene or a tumour suppressor gene (Refs 147–150). It has been recently revealed that IL-10 is highly epigenetically regulated (Refs 93, 151). It is worth noting that such a level of post-transcriptional regulation of IL-10 expression might be a relevant explanation for the differential expression and effects of IL-10 in different cells at the TME despite the existence of common pathways for IL-10 induction as previously mentioned in this review, via the action of non-coding RNAs including microRNAs (miRNAs) (Refs 152, 153), long non-coding RNAs (lncRNAs) (Refs 154–156), and circular RNAs (circRNAs) (Refs 144, 156, 157).

Epigenetic modulation of IL-10 on the post-transcriptional has been highly evident in several reports via DNA methylation, histone modifications and histone acetylation, which have been extensively studied before in several studies (Refs 158, 159) and recently reviewed in (Ref. 158). However, the epigenetic regulation of IL-10 via ncRNAs, miRNAs, lncRNAs and circRNAs is recently being explored. Therefore, a closer approach to exploring the epigenetic regulation of IL-10 via ncRNAs could aid in understanding the complex nature of this cytokine.

#### microRNAs (miRNAs) regulating IL-10

miRNAs are short ncRNAs around 18–25 nucleotides long that widely exist in plants, viruses and animals (Refs 29, 150, 160, 161). These miRNAs can regulate gene expression by either degrading the mRNA target or by suppressing mRNA translation and reducing mRNA stability by binding to the 3′UTR (untranslated region) of a target gene (Refs 125, 153). Thus, a miRNA could therefore inhibit or activate the expression of tumour suppressors or oncogenes. Generally, oncogenic miRNAs (oncomiRs) are found to be over-expressed in cancers, whereas miRNAs with tumour-suppressive function are found to be under-expressed (Refs 124, 146, 150). When these oncomiRs or tumour suppressor miRNAs are inhibited or stimulated, respectively, cancer cell metastasis, proliferation and survival may be reduced, depending on the specific miRNA being affected and the type of cancer (Refs 28, 29, 133). Moreover, some cancers are dependent on specific oncomiRs, and suppressing such oncomiRs could completely regress cancer growth (Refs 149, 151, 162).

Few studies have presented miRNAs that could modulate IL-10 expression. In a study, testing for the possible post-transcriptional modulation of IL-10R*α* and IL-10R*β* expression by miRNAs, three miRNAs were shown to have seed regions that target the 3′UTR of IL-10R*α*; miR-15a, miR-185 and miR-211. These miRNAs were shown to inhibit the proliferation of IL-10-treated melanoma cells, while their inhibitors caused an increase in cell proliferation in melanoma (Ref. 163). IL-10 was also shown to be targeted by several other miRNAs (Ref. 164). Another study showed that miR-106a could bind to the 3′UTR of IL-10 and significantly downregulate its expression *in-vitro* (Ref. 165). Two transcription factors; early growth response 1 (Egr1) and Sp1 were implicated in the induction of miR-106a, which consequently reduced IL-10 levels (Ref. 164). Furthermore, an inverse relation was reported between Egr1-stimulated miR-106a and IL-10 levels. It is also worth mentioning that miR-106a is part of a cluster that is known to be dysregulated in 46% of human T-cell leukaemias. Thus, it was deduced that the promotion of leukaemic cell survival by IL-10 might be through its modulation via miR-106a (Ref. 164).

Another miRNA reported to positively regulate IL-10 was miRNA-4661. The miR-4661 binding to the 3′UTR of IL-10 resulted in a net increase in the half-life of IL-10. This action was favoured by preventing tristetraprolin (TTP) from binding to the IL-10 mRNA (Ref. 166). TTP is an RNA binding protein that plays a critical role in regulating proinflammatory immune responses by destabilizing target mRNAs via binding to their AU-rich elements (AREs) in the 3′-UTRs of mRNAs (Ref. 167). Moreover, miRNA/IL-10 interactions were reported in a study by Liu *et al*. revealing that miR-98-mediated post-transcriptional control could potentially be involved in fine-tuning IL-10 production in endotoxin tolerance (Refs 168, 169). On the other hand, IL-10 was reported to upregulate miRNAs that contribute towards an anti-inflammatory response such as miR-187 or downregulate those that are highly pro-inflammatory, such as miR-155 (Ref. 164). IL-10 was able to downregulate the induction of miR-155 induced by LPS (Ref. 170). Moreover, *in-vivo* studies on mice deficient in miR-155, could not generate a protective immune response (Ref. 171). Whereas in IL-10 mice-deficient cells, miR-155 levels were shown to highly increase. It was previously known that miR-155 could target a number of genes involved in the immune response, such as suppressor of cytokine signalling (SOCS), inhibitor of NK-*κ*B kinase subunit epsilon (IKBKE) and Fas-associated death domain (FADD). Thus, targeting this miRNA by IL-10 is likely to elucidate key mechanisms through which IL-10 exerts control in the cell. Another study uncovered details of the IL-10 pathway by examining the effect of IL-10 on miRNAs, using IL-10 deficient mice for expression. Ten miRNAs were found to be upregulated in IL-10 deficient mice (miR-19a, miR-21, miR-31, miR-101, miR-223, miR-326, miR-142-3p, miR-142-5p, miR-146a and miR-155) (Ref. 172). miR-223 could hinder Roquin ubiquitin ligase by binding to its 3′UTR, eventually regulating IL-17 production and its inhibitor IL-10. Thus, this suggested a mechanism by which IL-10 could modulate the expression of IL-17 through miR-223. As previously mentioned, IL-10 can also induce the expression of anti-inflammatory miRNAs, such as miR-198 which is known to suppress TNF-*α* and IL-6. Consequently, this resulted in the promotion of an anti-inflammatory environment (Ref. 173). Collectively, such interesting findings of the mutual interaction between IL-10 and miRNAs discussed in the previous section highlighted an important role in the miRNA-mediated regulation of IL-10 expression and provided new insights into the intertwined mechanistic details of such immunomodulatory cytokine.

#### LncRNAs regulating IL-10

Long transcripts of RNA having more than 200 nucleotides, and not involved in protein translation are regarded as lncRNAs (Refs 16, 18, 154). LncRNAs play a significant role in the occurrence and development of cancer and thus, regulate the expression of cytokines such as IL-10 and IFN-*γ* as reported in a study by Tang *et al*. on non-small cell lung cancer (NSCLC) (Ref. 174). A large number of lncRNAs has been associated with cancer as recognized by genome-wide association studies on numerous tumours (Ref. 126). They are believed to exhibit functions such as tumour suppression and promotion, hence depicting to have a promising novel approach as biomarkers and therapeutic targets for cancers (Ref. 175). An increased expression of lncRNA SNHGI in cancerous breast cells of CD4 + TILs was also reported, whereas the expression of FOX and IL-10 was seen to be greatly reduced by siRNA SNHGI (Ref. 176). Moreover, silencing the lncRNA cox-2 was believed to increase the expression of IL-10, Arg-1 and Fizz-1 in M2 macrophages (Ref. 177). A study conducted by Zhou *et al*. reported reduced expression of IL-10 via suppression of lnc-LINC00473 (Ref. 178). Additionally, increased expression of IL-10 has been associated with the knockdown of lncRNA growth arrest-specific transcript 5 (GAS5) and reduced CRC cell proliferation while knockout of GAS5 promoted CRC colony formation and proliferation (Ref. 179). LncRNAs are known to regulate various signalling pathways such as TGF-*β*, STAT3, Hippo, EGF, Wnt, PI3 K/AKT and p53, whilst IL-10 is mostly involved in T-cell immune surveillance and suppression of cancer-associated inflammation. The expression of interleukins is regulated by lncRNAs that are known to be involved in various types of cancer. For instance, previous work by our group highlighted the potential of miRNA and lncRNA in the regulation of IL-10 in breast cancer, where miR-17-5p was identified as a dual regulator of TNF-*α* and IL-10. Additionally, knocking down the lncRNAs MALAT1 and/or H19 induced miR-17-5p and decreased TNF-*α* and IL-10 expression levels (Ref. 8). Such reports ed the immune-activator potential of miRNAs and the oncogenic potential of lncRNAs in cancers by regulating immunological targets in the TME. Hence, the extensive research on the relationship between the lncRNAs regulating IL-10 in various cancer needs to be validated further to establish a valid therapeutic link (Ref. 180).

#### CircRNAs regulating IL-10

CircRNAs are recognized as special ncRNA molecules with a distinctive ring structure and play significant roles as gene regulators and are considered one of the recently discovered epigenetic factors (Refs 153, 157). Abnormal production of circRNAs was found to influence the onset, progression and metastasis of cancer by acting as either tumour-suppressive or oncogenic factors (Refs 152, 181–183). This happens via interactions with proteins, miRNA sponge function and posttranscriptional regulation (Refs 155, 157, 184). Moreover, a line of evidence showed that circRNAs play pivotal roles in the chemoresistance (Refs 157, 185). Recently, specific circRNAs were found to possess an immunomodulatory function and alter the response of the TME by regulating the functions of tumour-infiltrating immune cells. For instance, CD4 + T cells activity is enhanced by circ0005519 through promoting the expression of IL-13 and IL-6 via affecting the expression of hsa-let-7a-5p (Ref. 186). On the other hand, circNT5C2 could attenuate the immune response by targeting miR-448 and serve as an oncogene via promoting tumour proliferation and metastasis (Ref. 187).

Since IL-10 function represents an unresolved enigma in cancer therapy, and since circRNAs also have dual roles in cancer therapy, the comprehensive understanding of circRNAs regulating IL-10 expression and function might be the key to answering numerous questions. Therefore, several studies that shed the light on novel circRNAs regulating IL-10 in different oncological and non-oncological contexts are highlighted. Some circRNAs can either enhance or inhibit IL-10 production and consequently could either promote or inhibit carcinogenesis. For example, circMERTK was reported to inhibit IL-10 production in colorectal cancer. The same study came to the conclusion that circMERTK knockdown reduced the activity of CD8 + T cells, suggesting that circMERTK may affect immunosuppressive activity through the circMERTK/miR-125a-3p/IL-10 axis (Ref. 188). According to another *in vitro* study, the downregulation of secreted PD-L1 by non-small cell lung cancer cells upon knockdown of circCPA4 resulted in the activation of CD8 + T cells in the TME (Ref. 188). In addition, the study found that PD-L1 abrogation reduced the expression of IL-10 in CD8 + T cells (Ref. 189). Circ103516 expression was found to be inversely correlated with IL-10 in inflammatory bowel diseases and thus it was postulated to play a proinflammatory role by sponging miR-19b. Additionally, it was discovered that circRNA HECTD1 contributed to the development of acute ischaemic stroke and that it was inversely linked with IL-10 production, suggesting that IL-10 played a protective function in acute ischaemic stroke (Ref. 190). In another cardiac context, the synthesis of IL-10 was decreased as a result of the overexpression of circFoxo3, a circRNA that is crucial in avoiding cardiac dysfunction brought on by myocardial infarction (Ref. 191). Downregulation of circ00074854 was reported to prevent polarization of M2 macrophages, which consequently alleviated the invasion and migration of hepatocellular carcinoma cells. According to the same study, macrophages exposed to exosomes produced by HepG2 cells that contained lower amounts of circ00074854 had significantly lower levels of IL-10 than those exposed to exosomes produced by HepG2 cells, demonstrating the direct relationship between Circ00074854 and IL-10 in different cancer settings (Ref. 192). Furthermore, a recent study emphasized the potential of CircSnx5 as a therapeutic target for immunological disorders since it has the ability to regulate the immunity and tolerance induced by DCs. It is interesting to note that knockdown of CircSnx5 led to a significant drop in IL-10, whilst overexpression of CircSnx5 was found to block DC maturation and boost IL-10 expression (Ref. 193). Another study focused on Circ0001598 as a potential target for treating breast cancer. It was discovered that circ0001598 regulates miR-1184 and PD-L1 via significantly increasing breast cancer proliferation, chemo-resistance and escape from immune surveillance. According to the same study mentioned above, depletion of circ0001598 increased breast cancer cells' susceptibility to Tratuzumab-induced CD8 + T cell cytotoxicity while decreasing the production of IL-10 (Ref. 194). Another study showed that the knockdown of circRNA PLCE1 ablated IL-10 production from macrophages while PLCE1 encouraged the transformation of epithelial cells into mesenchymal tissue, thus aiding glycolysis in colorectal cancer (Ref. 195). Another recently identified circRNA; circZNF609 has been linked to the pathogenesis of coronary artery disease, and forced overexpression of circZNF609 resulted in augmenting IL-10 expression (Ref. 196). It is also worth mentioning that a recent study discovered that circRNA NF1-419 attenuated inflammatory factors such as IL-10 and aging markers to postpone the onset of senile dementia (Ref. 197). Also, circGFRA1 has been indicated as a potential therapeutic target in prostate cancer; where Meng *et al*. reported that through a reduction in IL-10, knocking down circGFRA1 lessens the tumourigenic and immune-evading characteristics of prostate cancer cells (Ref. 198). Zhang *et al*. also discovered the role of circ0005075 in mediating neuroinflammation where silencing of circ0005075 in rat models resulted in a decrease in IL-10 production and protected against neuro-inflammation (Ref. 199). Another *in vitro* study revealed that circCdr1 overexpression enhanced the transcription of IL-10 both in naïve and pro-inflammatory macrophages (Ref. 200). CircCHST15 was recently reported to possess an oncogenic role by promoting immune escape through upregulating the expression of IL-10 and a sponging effect on miR-155 and miR-194 in lung cancer (Ref. 201). Additionally, circ_0046523 was found to promote carcinogenesis, mediate immunosuppression and abrogate CD8 + T cells function in pancreatic cancer via enhancing the secretion of IL-10 and TGF-*β* (Ref. 202). Furthermore, silencing circDNMT3B was discovered to decrease cell survival, promote apoptosis and increase IL-10 production in rat intestinal tissue (Ref. 203).

Collectively, it is quite clear that the circRNAs that inhibit IL-10 production from tumour cells act as tumour suppressors, while those that increase the production of IL-10 from tumour cells promote oncogenesis, cell survival, drug resistance and mediate immunosuppression. This highlights the promising role of such circRNAs as novel immunotherapeutic molecules that could ablate IL-10 production and act as a powerful immunomodulatory anti-cancer treatment for several cancer patients.

### Pharmacogenomic approach: single nucleotide polymorphisms in IL-10 and its receptor

#### IL-10 gene

A very important basis for studies and research in IL-10 regulation is the examination of its genomic location and promoter structure. IL-10 gene encodes a protein, 178 amino acids long, which is secreted after cleavage to be comprised of 18 amino acids (Ref. 54). At the proximal promoter sequence of IL-10 in the human genome, there is a TATA box located upstream of the translation start site, for several transcription family members, including nuclear factor-*κ*B (NF-*κ*B), STAT, specificity protein (Sp), CREB, CCATT enhancer/binding protein (C/EBP), c-musculoaponeurotic fibrosarcoma factor (c-MAF), which have been characterized as ‘critical’ factors in regulating IL-10 expression (Ref. 204).

#### IL-10 signalling

Next, it is necessary to understand how IL-10 can signal through its receptor. IL-10R is a heterodimeric receptor complex composed of two chains (IL-10R*α* ‘R1’ and IL-10R*β* ‘R2’). The *α*-chain binds directly to IL-10, while the *β*-chain is subsequently recruited into the IL-10/IL-10R*α* complex (Ref. 205). The binding of IL-10 to IL-10R*α* induces a conformational change in the receptor, allowing it to dimerize with IL-10R*β*. This dimerization leads to signal transduction in target cells (Ref. 206). When the IL-10 complex is formed, tyrosine kinases Tyk2 and Jak1 become activated and phosphorylate specific tyrosine residues. This phosphorylation further activates the cytoplasmic inactive transcription factor; STAT-3 resulting in the translocation and transcriptional activation (Ref. 207). IL-10 rapidly activates STAT-3 and remains phosphorylated over a sustained period, unlike the transient phosphorylation of IL-6 (Ref. 208). The STAT-3 docking sites in IL-10R1 appear to be sufficient to induce IL-10-mediated proliferative responses (Ref. 209). While IL-10R2 intracellular domain seems to provide the docking site for Tyk2. Thus, most IL-10-specific cellular functions appear to reside in the IL-10R1 chain, whereas IL-10R2 recruits the downstream signalling kinases (Ref. 210).

#### SNPs affecting IL-10

The *IL-10* gene promoter and IL-10R have been found to include a significant number of SNPs (Refs 145, 211). There is strong evidence that several of these polymorphisms are linked to the differential expression of IL-10 *in vitro* and in some situations, *in vivo* (Refs 161, 212, 213). Some of these IL-10 variants have been associated with either low or high expression in several cancer types. For example, some genotypes have been evidenced to be correlated with a decreased expression of IL-10 and a higher risk to develop prostate cancer or non-Hodgkin's lymphoma (Refs 214, 215). On the other hand, other evidence concluded that some IL-10 variants are associated with higher expression of IL-10 and consequently, an elevated risk for cancer development of multiple myeloma, cervical cancer and gastric cancer in patients harbouring a particular IL-10 variant (Refs 216–218). Also, it has been demonstrated that the IL-10 gene transcription and translation were impacted by the SNPs in the IL-10 promoter region, leading to aberrant cell division and emergence of breast cancer (Ref. 219). Table 2 summarizes most of the IL-10 polymorphisms documented in the literature and their association with cancer development and risk.

**Table 2.** IL-10 polymorphisms and their association with cancer development and risk

Type of cancer	IL-10 polymorphism	Variant	Contribution	References
Chronic lymphocytic leukaemia (CLL)	−1082	1082 G/A and A/A	Increased risk to CLL	(Ref. 220)
Prostate cancer	−1082	−1082 AA	High risk factor and susceptibility	(Ref. 221)
Cervical cancer	rs1800896	AG/AA genotypes	High risk factor and susceptibility	(Ref. 222)
Breast cancer	rs1800896 rs1800871 rs1800872	AA genotypes	High risk factor and susceptibility	(Ref. 223)
Gastric carcinoma	−1082, −592 −819	GCC, ATA, AG haplotype	Advanced stage, high risk factor and susceptibility,	(Ref. 224)
Oral cancer	−1082	−1082 G allele	High susceptibility to oral carcinoma	(Ref. 225)
Multiple myeloma	IL-10G IL-10R	IL-10 G 136/136, IL-10R 112/114	Increased susceptibility	(Ref. 226)
Lung cancer	−592	A > C	Increased risk	(Ref. 227)
Non-Hodgkin's lymphoma	−1082 −592 −819	−1082 AA, ATA, ACC haplotypes	More aggressive form of the disease.	(Ref. 228)
Acute lymphoblastic leukaemia	−1082	−1082 GG	Low possibility of poor response to prednisolone	(Ref. 229)
Acute myeloid leukaemia	−819	−819 T/C	Increased risk of AML	(Ref. 230)
Colorectal cancer	−819	T > C genotypes	Increased risk of colorectal cancer	(Ref. 231)
Cutaneous malignant melanoma	−1082 −592 −819	High risk, larger tumour thickness, disease progression and shorter survival	1082 AA, 1082 GG, ACC/ACC, ACC/ATA, ATA/ATA	(Refs 232, 233)

Since IL-10 has a role in malignancy, it is regarded to be the subject of numerous disputes in the literature, whether it has a positive or negative effect. As a result, whether IL-10 blockage is effective as an immunotherapeutic strategy is another unsolved puzzle. This opens the door to a crucial query that might provide the answer. However, it has not yet been addressed in the literature. It remains unclear whether SNPs in the IL-10 or its receptor account for the varying effects of IL-10 inhibition on cancer treatment. A clinical investigation addressing the existence of SNPs in IL-10 or its receptors and their impact on the response to IL-10 therapy is necessary. These pharmacogenomic investigations will aid in the development of immunotherapeutic modalities by identifying the most qualified individuals to provide these cutting-edge drugs.

## Conclusions

**Figure 3.**
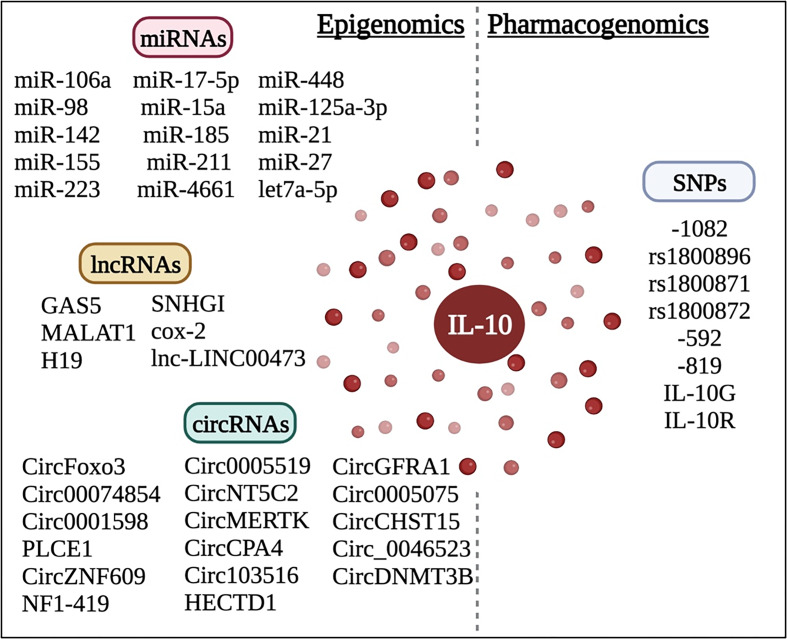
Epigenomic and pharmacogenomic regulation of IL-10 in oncology

This review highlighted the controversial functions of IL-10 in oncology. Such contradictory information prevented researchers from determining whether exogenous IL-10 administration or blockage will boost the immune system and combat changes at the TME. This could be explained by the fact that IL-10 has two distinct functions depending on which immune cell and which receptor would be activated. Also, epigenetic regulation of IL-10 in cancer via ncRNAs is quite complex (Fig. 3). Also, the relationship between IL-10 SNPs will help us better understand the precise function of IL-10 in the TME and will help us develop more individualized immunotherapeutic approaches by classifying patients into responders and non-responders.
